# Mothers’ experiences of feeding their premature infants after discharge from the hospital: a systematic review and thematic synthesis

**DOI:** 10.1186/s13006-026-00893-y

**Published:** 2026-09-09

**Authors:** Sonja Jacobs, Deanna Gibbs, Christina Smith, Kathy Hui, Nathan Davies

**Affiliations:** 1 Barts Health NHS Trust, London, UK; 2 Centre for Psychiatry and Mental Health, Wolfson Institute of Population Health, Queen Mary University of London, London, UK; 3 Division of Psychology and Language Sciences, University College London, London, UK; 4 University College London Hospital, London, UK; 5 Centre for Genomics and Child Health, Blizzard Institute, Queen Mary University of London, London, UK

**Keywords:** Premature, Feeding, Agency, Experience, Cue-based, Mind-mindedness

## Abstract

Being born prematurely has profound implications for feeding, which is one of the most complex and developmentally significant tasks in the first year of life. Various factors influence feeding outcomes and experiences including fatigue and coordination difficulties associated with prematurity, heightened parental anxiety, and altered parent-infant interaction that can result in maladaptive mealtime routines and behaviours. The transition from hospital to home is a sensitive period for parents of premature infants. This review aims to understand parents’ experiences of feeding their premature infants in the first year after discharge. This synthesis will inform the development of effective post-discharge support strategies and guide future research. A systematic search of six databases (MEDLINE via PubMed, CINAHL, EMBASE, AMED, PsycINFO, and Web of Science) was conducted. Qualitative studies published between 2000 and 2025 that describe parents’ experiences of feeding their premature infants after hospital discharge were quality-appraised and analysed using thematic synthesis. Twenty-two studies conducted across 10 countries were included. All studies focused on mothers of premature infants across all levels of prematurity and most centred on breastfeeding experiences. Four main themes illustrate that feeding experiences are shaped by personal, institutional, and societal influences and that mothers across diverse countries describe remarkably similar experiences despite variations in healthcare systems and models of care. The main themes were the complex reality of the feeding journey, negotiating agency in the feeding journey, navigating the institutional landscape and the impact of societal norms on feeding experiences. The review showed that the struggle to build and sustain maternal agency and confidence in feeding their premature infants, does not end at discharge; for many, it intensifies at home. Supporting parents of premature infants requires attention not only to feeding techniques but also to the relational, institutional, and societal forces that shape maternal agency and confidence long after hospital discharge.

**Supplementary Information:**

The online version contains supplementary material available at https://doi.org/10.1186/s13006-026-00893-y.

## Background

It is estimated that internationally 13.4 million infants were born prematurely in 2020, accounting for 9.9% of all births [1]. Prematurity, defined as birth before 37 weeks, is categorised as extremely preterm (less than 28 weeks gestation), very preterm (28 to less than 32 weeks), or moderate-to-late preterm (32 to 36 + 6 weeks) [2]. Being born early profoundly shapes early feeding, a complex task requiring physiological maturity, caregiver responsiveness, and supportive environments.

More than 40% of ‘healthy’ premature infants are reported to have feeding difficulties in their first year, with difficulties persisting beyond age four for many [3, 4]. Early infant experiences, disruptions to normal parenting and parental mental health directly shape feeding interactions, responsiveness, and the infant’s feeding skills [5–9]. Premature infants often have limited parental contact, sensory overstimulation, and pain during medical procedures while in hospital [5, 8]. Hospitalisation disrupts normal parenting and bonding through separation, altered parental roles, reduced involvement in care, and emotional strain [8, 9]. Furthermore, these early disruptions can heighten parental concerns and anxiety about feeding and eating behaviour and lead to more controlling feeding practices.

Feeding is a relational, dyadic process [10] and foundational to the feeding relationship is mothers’ abilities to see their infants as individuals with their own minds, feelings and intentions, known as mind-mindedness [7]. Mind-mindedness encompasses recognising, interpreting, and responding to infants’ internal states, e.g. intentions, emotions and preferences [11]. Mothers who show mind-mindedness when their premature infants are six months corrected age have more positive feeding interactions at 12 months corrected age [6, 7]. Cue-based feeding is a practical and observable expression of mind-mindedness and is relevant to understanding relational feeding processes in premature infants. Cue-based feeding, where the feeder observes the infant’s behaviours and cues and adjusts their behaviour accordingly, improve feeding outcomes in neonatal settings [12–15]. Interpreting and responding to infants’ cues can however become challenging after discharge, when support is limited and when dealing with the trauma of premature birth [6, 12]. Over time, difficulties recognising and responding to feeding cues can lead to maladaptive mealtime dynamics and may contribute to food aversion, disengagement from meals, and poorer growth outcomes [16].

The transition home from hospital is a sensitive period for parents. Many parents feel anxious about taking their infant home [17], with feeding and growth among their main concerns [12]. It is difficult to predict which infants will require ongoing support after discharge [18]. Parents of infants considered low risk at discharge often report high support needs, possibly due to their limited connection with specialist services [19]. The parental trauma of early birth, including symptoms of depression, anxiety and post-traumatic stress, may only become visible after discharge [12, 14, 20].

Post-discharge support can reduce parental stress and improve maternal agency in caring for their infant [21, 22]. Maternal agency refers to mothers’ perceived capacity to act intentionally and confidently when feeding and caring for their premature infant, this includes recognising cues, making informed decisions, and navigating relational and institutional influences [22]. However, a multi-country European study reported that post-discharge support was mostly insufficient [23]. Understanding the emotional, practical, and informational challenges of feeding premature infants after discharge is crucial for developing targeted interventions that support families and improve their long-term outcomes.

This review aimed to understand how parents experience feeding their premature infants in the first twelve months after discharge. Through a systematic review and thematic synthesis, this study explored the factors that shape feeding experiences to inform future research and support strategies.

## Methods

### Design

This qualitative systematic review used thematic synthesis to combine findings from qualitative studies to move beyond the original findings and present a novel understanding of the data [24]. This review is reported in accordance with the Preferred Reporting Items for Systematic Reviews (PRISMA) [25, 26] guidelines to ensure transparency in the search, screening, and study selection processes. The Enhancing Transparency in Reporting the Synthesis of Qualitative Research (ENTREQ) checklist [27] was used to support the comprehensive and rigorous reporting of the qualitative synthesis methods. The study protocol was registered in the PROSPERO International Prospective Register of Systematic Reviews (ID 646786, registered on 3/3/2025).

### Eligibility criteria

Studies were eligible if they explored parents’ experiences of feeding their premature infants during the first year after discharge from hospital. Premature infants were defined as those born before 37 weeks gestation, with no minimum birthweight requirement. Infants could have been discharged directly home with their mothers or after a period of neonatal care. Eligible study designs included qualitative or mixed-methods that reported parental experiences included first-hand accounts of challenges, emotional responses, practical strategies and support received or needed in breast, bottle or gavage feeding. Studies were excluded if they focussed solely on in‑hospital feeding, involved term infants or infants with unrelated medical conditions, practical management of mother’s own milk such as expression, storage or managing donated milk or infant nutrition and growth.

### Search strategy

The SPIDER search strategy guided the generation of key concepts and search terms [28] (Table 1). An exhaustive three-step search strategy was developed by the research team in conjunction with an expert librarian. The first, completed during the development of the proposal, included an initial limited search of Medline and CINAHL to identify relevant articles and develop a full search strategy. In the second step the search strategy was reviewed by a librarian using the Peer Review of Electronic Search Strategies (PRESS) 2015 guidelines [29] and used to search databases, including MEDLINE via PubMed, CINAHL, EMBASE, AMED, PsycINFO, and Web of Science. The search strategy was adapted for each database and information source. The final search was conducted on 12/03/2025. The full search strategy is provided in additional file 1. During the final step, the reference list of all included sources was screened for additional sources.

**Table 1:** Text words, index terms, and subject headings identified for the full search

Key concept	Text words/Index terms/MeSH terms (Medical Subject Headings)
Feeding	Bottle Feeding; *Breast feeding; *Feeding Behaviour; weaning; eating; complementary feeding; feeding difficulties; cue-based feeding; infant-driven feeding
Premature infants	Infant, Low Birth Weight/or exp Infant, Very Low Birth Weight/or exp Infant, Premature/ premie*; preemie*; premature birth preterm; premature; “extremely preterm”; “extremely premature”
Parents	exp Parents/or Caregivers/ mother*; father*; parent*; caregiver*; guardian* Mother-child relations; mother-infant relations; parent stress; parent confidence; parent attunement, parent self-efficacy
Post-discharge	Patient Discharge/; Aftercare/; discharge; after discharge; transition, post-discharge, follow-up care

This search strategy identified all relevant peer-reviewed, full-text qualitative and mixed-methods studies in which qualitative findings were reported separately. Non-empirical publications such as letters, commentaries, editorials, reviews, and conference abstracts without full texts were excluded. Studies reporting mixed data, for instance, where some of the participants or the context met the inclusion criteria, were included if the results relevant to this review were reported separately or if more than 70% of the data met the inclusion criteria for this study. The search was limited to studies published since 2000, ensuring that the synthesis reflected data since significant advancements in the medical and neuroprotective care of premature infants [8]. Only articles published in English or those for which journals provided English translations, were included due to feasibility and to minimise the risk of misinterpretation. Titles and abstracts in other languages were translated using Microsoft Copilot, and exclusion of potentially relevant non-English studies were documented at full-text stage to reduce language bias.

### Study selection

Records identified through the search strategies were imported into Covidence systematic review software [30], where duplicates were automatically removed and reports were screened and selected. The first author (SJ) independently screened all titles and abstracts against the inclusion and exclusion criteria, followed by full-text screening of all the retrieved reports. The review protocol determined that 20% of titles, abstracts and full texts will be reviewed by a second reviewer to balance rigour with feasibility in terms of resources [24, 25], allowing for the assessment of agreement and identification of discrepancies, The study selection process was piloted on a small number of studies resulting in the second reviewer (KH) independently screening 30% of the titles and abstracts, followed by 35% of the selected full-text reports. Where necessary, consensus on inclusion was reached through discussion between the first and second reviewers or through discussion with the other authors.

### Quality appraisal and data extraction

Quality appraisal and data extraction occurred simultaneously. Eligible studies were imported into NVivo v15 [31], to support data extraction and qualitative synthesis [32]. A multidimensional approach was taken to assess both the quality of reporting and the conceptual depth and breadth of the included studies, ensuring the credibility of this review. The Critical Appraisal Skills Programme (CASP) Qualitative Studies Checklist [33] was used to evaluate methodological quality. The Richness and Thickness Assessment Tool was used to appraise the relevance, conceptual depth and breadth of the included studies [34]. A customised form was created in NVivo for data extraction, containing the metrics of both quality appraisal tools and study characteristics.

The first author (SJ) independently conducted all appraisals, and a second reviewer (KH) independently completed the CASP assessments for 30% of the included studies to enhance rigour. Consistent with recommendations to avoid excluding studies that may offer valuable insight despite methodological limitations [35], no studies were excluded on the basis of appraisal outcomes. Instead, studies were categorised from high to low quality for methodological rigour and conceptual contribution. The lower-quality studies did not generate new codes but were used to confirm and support existing codes.

### Data synthesis and quality assessment

Qualitative data were analysed using thematic synthesis, which involved generating analytic themes across studies rather than providing narrative summaries of each individual study [24]. Synthesis was an iterative and inductive process that started with the development of descriptive codes generated through line-by-line coding to capture key concepts. The codes were then organised into themes and subthemes [24]. Although gestational age and feeding method may shape feeding experiences, these variables were not used as analytic categories because thematic synthesis focuses on shared experiential patterns across studies rather than stratifying findings by participant characteristics. In addition, these details were not consistently reported across the included studies.

Coding and synthesis were conducted by the primary author (SJ) and included regular discussion and reflection with the review team. Through these discussions, emerging codes and themes were explored alongside reflection on the nuanced meaning of words, concepts and experiences. Confidence in each synthesised finding was assessed using the GRADE-CERQual approach [36, 37].

## Finding

### Study characteristics

**Fig. 1:**
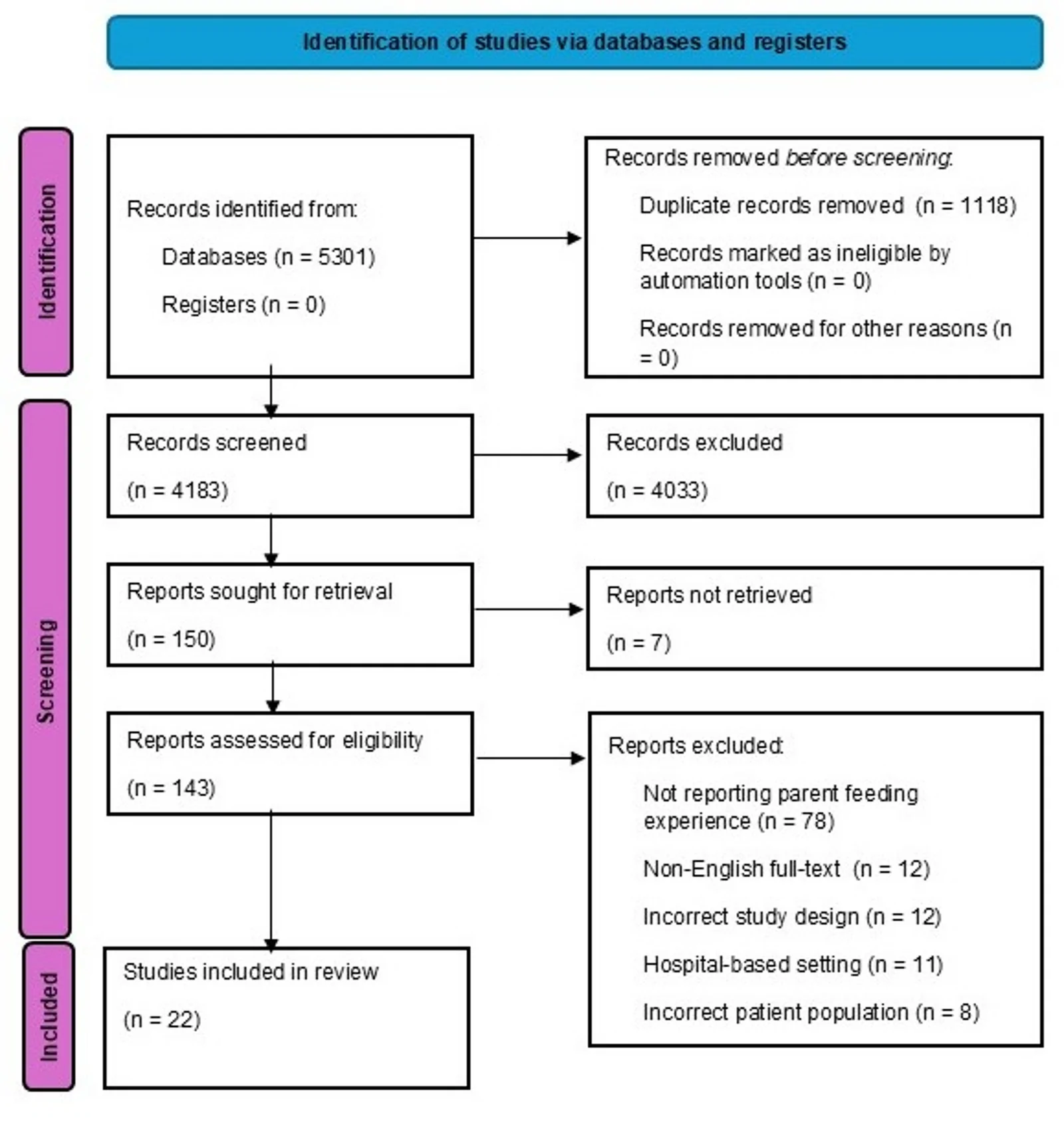
PRISMA flow diagram. (adapted from “The PRISMA 2020 statement: An updated guideline for reporting systematic reviews,” by Page et al., 2021, PLOS Medicine, 18[3], e1003583. CC BY 4.0 [26]).

The study selection process is reported on the PRISMA [25, 26] flow diagram (Fig. 1). Twenty-two studies published between 2000 and 2024 were included in this synthesis (Table 2). The studies spanned multiple continents and a range of economic contexts. High-income settings were represented by studies from the United States [38–42], Canada [43–46], Sweden [47–50], Finland [51, 52], Norway [53] and Iceland [54]. Middle-income contexts were reflected in studies from China [55], Brazil [56] and Columbia [57], whereas two studies were conducted in Iran [58, 59], representing a lower-middle-income setting [60]. There were no studies conducted in low-income countries.

**Table 2:** Study characteristics

First author	Publication year	Country	Research aim or question	Infant feeding method under investigation	Gestational age (in weeks)	Data collection time points	Sample size	Participants
Breivold [53]	2019	Norway	To explore mothers’ experiences after coming home from the hospital with a moderately to late preterm infant	Breast, Bottle, Mixed, Tube	28–36	2–3 months post-discharge	10	Mothers
Brown [38]	2013	United States	To describe the change that occurs in a mother’s IWM of feeding from the time feedings begin in the NICU until 2 weeks after hospital discharge.	Breast, Bottle, Mixed	< 37	2 weeks post-discharge	12	Mothers
Callen [43]	2005	Canada	To determine the barriers to the successful establishment and maintenance of breastfeeding in VLBW infants during hospitalisation in a tertiary care setting, after discharge home, and at 1 month, 3 months, 6 months, and 12 months corrected age.	Breastfeeding	28–36	3, 6, 12 months post-discharge	59	Mothers
Dosani [44]	2017	Canada	What is the mother’s experience of caring for LPIs with respect to breastfeeding?	Breastfeeding	34–36 + 6w	6–8 weeks post-birth	11	Mothers
Ericson [48]	2020	Sweden	To describe the cessation of breast-feeding in mothers of preterm infants up to 12 months after birth.	Breastfeeding	< 37	8w after discharge, 6 m and 12 m postnatal	149	Mothers
Ericson [47]	2017	Sweden	To describe mothers’ experiences of proactive and reactive breastfeeding telephone support after discharge from a NICU.	Breastfeeding	< 37	2–6 m post-discharge	26	Mothers
Espitia [57]	2013	Columbia	To describe experiences of mothers caring for their preterm offspring on the first day after being discharged from the hospital.	Not specifically stated	28–36	Unclear, soon after discharge	10	Mothers
Flacking [49]	2007	Sweden	To determine how mothers of very preterm infants experience the process of becoming a mother and breastfeeding after the discharge from the NU.	Breastfeeding	< 32	3–12 m post-discharge	25	Mothers
Hemati [58]	2017	Iran	To investigate the challenges that mothers encounter after their infants’ discharge to gain comprehensive and in-depth knowledge on this issue.	Not specifically stated	28–36	Before discharge and at first follow-up	23	Mothers
Jiang [55]	2024	China	To explore breastfeeding experiences among mothers who were separated from their preterm infants post-delivery in Shanghai, China.	Breastfeeding	28–36	42-days postpartum	18	Mothers
Jonsdottir [54]	2022	Iceland	To describe the initiation and duration of any and exclusive breastfeeding at the breast for mothers of LPT twins and term twins during the first 4 months and to explore the breastfeeding experiences of mothers of LPT twins.	Breastfeeding	34–36 + 6w	First 6 months postnatal age	14	Mothers
Kair [39]	2015	United States	To examine the overall breastfeeding experience of mothers of late preterm infants as well as the reasons for weaning prior to 1 year.	Breastfeeding	34–36 + 6w	Weekly for a month, monthly for 12 months or until weaning from breastfeeding	44	Mothers
Kennedy [40]	2000	United States	To study the feeding experiences and nutritional issues of families with preterm infants.	Not specifically stated	< 35	12 m adjusted age	6	Mothers
Marigheti Brassarola [56]	2023	Brazil	To analyse the use of the WhatsApp^®^ application, as a technological tool, to help mothers in the post-discharge follow-up of their premature infant	Not specifically stated	< 37	Up to 6 m post-discharge	18	Mothers
Niela-Vilén [51]	2015	Finland	To describe the perceptions of breastfeeding	Breastfeeding	< 35	12 m adjusted age	22	Mothers
Niela-Vilén [52]	2019	Finland	To describe maternal emotions regarding and insights into breastfeeding during the first year after a preterm	Breastfeeding	< 35	3, 6, 12 months post-discharge	80	Mothers
Palmér [50]	2019	Sweden	To describe the breastfeeding experience of mothers of preterm infants from birth up until 12 months after birth.	Breast, Bottle, Mixed	< 37	At discharge, 8w, 6 m, 12 m postnatal age	270	Mothers
Parker [41]	2020	United States	To determine the array of factors that comprise the domains of the Theory of Planned Behaviour, attitudes, perceived control, and perceived subjective norms, related to breastfeeding among mothers of preterm infants	Breastfeeding	< 37	2–6 m post-discharge	23	Mothers
Premji [45]	2017	Canada	What does it mean to be a mother of a late preterm infant?	Not specifically stated	34–36 + 6w	14-34w post-natal	11	Mothers
Reyna [42]	2006	United States	To explore mothers’ perceptions of their experiences in feeding their preterm infants in the early weeks after hospital discharge.	Not specifically stated	< 32	2-3w post-discharge	27	Mothers
Valizadeh [59]	2014	Iran	To study and describe the experiences of mothers on feeding premature infants during the first week after discharge in a content-analysis qualitative study.	Breastfeeding	< 37	2–3 weeks post-discharge	14	Mothers
Wheeler [46]	2009	Canada	What factors are associated with early termination of breastfeeding	Breastfeeding	< 37	1w, 6w post-discharge	144	Mothers

Only mothers were participants in all the studies with sample sizes ranging from 6 to 270, including a total of 1016 mothers of premature infants. Eight studies recruited mothers of infants at any level of prematurity [38, 41, 47, 48, 50, 56, 59, 61], three under 35 weeks gestation [40, 51, 52], two under 32 weeks gestation [42, 49], five between 28 and 36 weeks gestation [43, 53, 55, 57, 58], and three studies between 34 and 36 weeks and 6 days gestation [39, 45, 54], with one of these focusing on late premature twins only [54]. Data were gathered at different time points between discharge and 12 months, with 15 studies using a single data collection time point and 7 having multiple episodes per participant.

Fourteen studies focused on breastfeeding after discharge and explored barriers [43], decision-making [41], cessation [46, 48], experiences [39, 44, 49, 50, 52, 54, 55, 59], and support [47, 51]. Of the remaining eight studies, six did not specify feeding type [38, 40, 43, 54–56] and two studies included breast, bottle and mixed feeding [36, 51]. Four of these studies explored feeding more broadly, including feeding experiences [42], decision making [40], post-discharge support [56] and the development of an internal working model of feeding [38]. Three studies explored feeding experiences within the wider context of bringing a premature infant home [53, 57, 58], and one explored experiences with community-based support [45] None of the studies specifically explored the introduction of solid foods.

### Quality assessment

Six review findings were developed from the themes and subthemes generated in the thematic synthesis (discussed in the next section) and represent the key interpretive conclusions of the review (Table 3). The four GRADE-CERQual components; methodological limitations, coherence, adequacy of data, and relevance [36, 37] were evaluated for each finding to determine an overall confidence rating. Across the review, three of the six findings were judged to have high confidence. Two finding were rated moderate confidence, and one low confidence, primarily due to the limited number of contributing studies and restricted contextual diversity. A summary of the CERQual assessment for each finding is presented in (Table 3) and more detailed assessment information is available in additional file 2.

**Table 3:** CERQual summary of qualitative findings

Synthesised review finding	Studies contributing to the finding	Overall CERQual Confidence	Explanation of CERQual assessment
1. Feeding a preterm infant in the early weeks is physically and emotionally demanding, shaped by intensive routines, exhaustion, and moments of connection.	[39–42, 44–59, 38]	High confidence	Supported by rich, consistent evidence with only minor concerns.
2. Breastfeeding is not simply a nutritional act or a technical skill but a deeply symbolic and relational practice through which mothers negotiated identity, responsibility, and the emotional aftermath of premature birth.	[39–42, 44–59, 38]	High confidence	Supported by rich, consistent evidence with only minor concerns about coherence, specifically about different descriptions and interpretations of identity
3. The meaning mothers assign to infant cues played a central role in shaping maternal agency, confidence, and the emotional tone of the feeding relationship.	[39–42, 44–46, 48–51, 53, 54, 56–59, 38]	Moderate confidence	Moderate concerns about coherence, specifically about different descriptions and interpretations of agency and confidence reduce confidence slightly.
4. Reframing feeding decisions involve redefining what it meant to be a ‘good mother’, shifting the focus from the method of feeding to the infant’s health, growth, the feeding relationship and the mothers’ own wellbeing.	[38–41, 46, 47, 49–54, 56, 57]	Moderate confidence	Moderate concerns about coherence and richness of descriptions, confidence therefore reduced.
5. Institutional structures and availability and adequacy of support shape not only feeding practices within the hospital but also mothers’ confidence and agency in decision-making and the feeding relationship long after discharge.	[38–45, 47, 49, 51–55]	High confidence	Supported by rich, consistent evidence with only minor concerns.
6. Societal messages of ‘good mothering’ and good feeding often fail to acknowledge the unique challenges associated with prematurity.	[49–52, 59]	Low confidence	Limited studies, mainly located in Scandinavian countries included data that contributed to this finding. Concerns about adequacy and relevance reduce confidence.

### Thematic synthesis

This synthesis examines how mothers of premature infants negotiate agency and confidence in feeding their premature infants in the home environment whilst also navigating institutional and societal influences. A socio-ecological lens, which conceptualises human behaviour as shaped by interacting individual, relational, organisational, and societal systems, provides a useful lense for understanding feeding as a dynamic, relational and context-dependent practice that evolves over time [62–64]. The first two themes focus on the individual and microsystem levels: the first theme describes the lived and embodied realities of feeding a premature infant at home, followed by a second theme that explores the intrapersonal and relational processes through which mothers make sense of these experiences and construct meaning, identity and agency. Themes three and four move outwards to the organisational and societal levels, illustrating how institutional structures and societal expectations shape, constrain, and sometimes amplify mothers’ efforts to make sense of feeding (Table 3). Together, these themes show how mothers work within, and sometimes push against, personal, relational, institutional, and societal forces as they strive to build agency and confidence in feeding their premature infant.

**Table 4:** Contribution of individual studies to themes and subthemes

Themes	The complex reality of the feeding journey		Negotiating agency in the feeding journey			Navigating the institutional landscape		Impact of societal norms on feeding experiences
Sub-themes	**Embodied labour and emotional turbulence**	**Moments of connection**,** learning**,** and emerging confidence**	**Maternal identity: Becoming the mother of a premature infant**	**Interpreting the infant: Making sense of cues and participation**	**Reframing feeding decisions: Negotiating expectations**	**Institutional routines and their lasting imprint on feeding**	**Professional guidance and support**	
Breivold (53)	x	x	x	x	x		x	
Brown (38)	x	x		x	x	x		
Callen (43)				x			x	
Dosani (44)		x	x	x		x		
Ericson (48)		x	x	x				
Ericson (47)		x	x		x	x	x	
Espitia (57)		x		x	x			
Flacking (49)	x	x	x	x	x	x		x
Hemati (58)		x	x	x				
Jiang (55)		x	x			x	x	
Jonsdottir (54)		x	x	x	x	x	x	
Kair (39)	x	x		x	x		x	
Kennedy (40)		x	x	x	x		x	
Marigheti Brassarola (56)	x		x	x	x			
Niela-Vilén (51)	x	x	x	x	x	x	x	x
Niela-Vilén (52)	x	x	x		x	x	x	x
Palmér (50)	x	x	x	x	x			x
Parker (41)	x	x	x	x	x	x	x	
Premji (45)		x	x	x			x	
Reyna (42)		x	x	x		x	x	
Valizadeh (59)		x	x	x				x
Wheeler (46)		x		x	x			

### The complex reality of the feeding journey

During the early weeks at home, feeding became the centre of daily life, confronting mothers of premature infants with a mix of challenges that shaped their emerging sense of agency. This theme captures the complex and often contradictory nature of the early feeding journey. It unfolded through two interwoven processes forming two subthemes: ‘embodied labour and emotional turbulence’ and ‘moments of connection, learning, and emerging confidence’ that helped mothers persevere. Together, these processes formed the shifting terrain on which the mother‒infant feeding relationship was negotiated.

### Embodied labour and emotional turbulence

The joy of bringing a premature infant home was often short-lived, quickly leading to feelings of anxiety, overwhelm, and exhaustion. The early weeks were described as physically and emotionally draining, largely due to the intensity of feeding routines that dominated day and night [38, 42, 46, 49, 50, 53, 54, 57]. Feeding--specific challenges were common, including persistent worries about milk production [39, 41, 44, 47–51, 53–56], infant weight gain [38, 41, 45, 51, 54, 56, 59], ongoing feeding difficulties associated with prematurity, such as poor coordination, low stamina and difficulty latching [38, 40–42, 44, 45, 50, 51, 54, 56–59], and maternal health issues such as mastitis [39, 48, 50, 51]. These pressures were compounded by the broader demands of caring for a medically vulnerable infant while managing household responsibilities and family life [42, 45, 53, 54, 59]. These challenges often left many mothers feeling depleted and unsure whether they were doing enough for their infants.

The pursuit of successful feeding often required significant physical and emotional labour, and breastfeeding in particular was described as exhausting, painful and all-consuming [49–53, 56]. Feeding was described as *“very frustrating”*, *“a lot of work*,*”* and *“exhausting”* by mothers in one study [44] and, in another study, as *“robotic”*, *“instrumental”* and like a *“military camp”* [54]. Feeding routines could easily shift from a nurturing activity to a rigid and regimented task that is emotionally draining and burdensome, particularly when mothers feel solely responsibility for their infant’s growth and wellbeing [42, 50, 51, 58, 59].

When breastfeeding was difficult or unsuccessful, mothers described profound guilt, shame, anxiety, sadness, and a sense of failing as a mother [39, 49–53]. These feelings impacted the mothers’ fragile sense of agency and confidence. However, even within this exhausting and emotionally turbulent landscape, mothers also described moments of connection and growing confidence.

### Moments of connection, learning, and emerging confidence

Despite hardship and sacrifice in the feeding journey, many mothers highlighted the rewards of breastfeeding, describing a sense of closeness, bonding and a special connection between the mother and infant [38, 39, 41, 50–52]. Feeding a premature infant, whether by breast or bottle, was described as a skill learnt and refined through close observation, ‘trial and error’ and a growing familiarity with feeding cues [38, 49, 53, 57, 58]. Recognising the ‘small wins’, for instance, a feed that went well, or recognising hunger cues, offered reassurance and a sense of coping amidst exhaustion [38, 50, 53, 54, 56]. These moments of success and progress were deeply valued and reinforced the mothers’ sense of competence and maternal identity and strengthened the feeding relationship [38, 41, 44, 45, 50–53].

In addition to maternal skill development and these restorative moments, mothers were reassured by observing their infants’ developing feeding skills, including improved coordination, wakefulness, endurance, and hunger cues [38, 42]. For some, these developments transformed their feeding experience from hard work to something they and the infant looked forward to [42].

Together these two subthemes illustrate how the early feeding journey was shaped by the dynamic interplay of strain and reward. While the physical and emotional demands of feeding a premature infant often left mothers feeling depleted and unsure of their competence, moments of connection, infant progress, and small successes offered powerful counterweights that nurtured their confidence and strengthened the developing relationship.

### Negotiating agency in the feeding journey

This theme captures the tension between mothers’ idealised expectations of feeding and the complex, often unpredictable realities of premature birth and hospital discharge. Across studies, breastfeeding was not only considered a nutritional act or a technical skill but a symbolic and relational practice through which mothers negotiated identity, responsibility, and the emotional aftermath of early delivery. Three sub-themes illustrate how mothers negotiated agency and confidence during the early months of their feeding journey: ‘maternal identity: becoming the mother of a premature infant’; ‘interpreting the infant: making sense of cues and participation’; and ‘reframing feeding decisions: negotiating expectations.’

### Maternal Identity: Becoming the mother of a premature infant

Although feeding a premature infant brought both challenges and rewards, many mothers internalised challenges as personal shortcomings. Mothers described feeling unprepared and blamed themselves for not meeting their own expectations of good motherhood [51]. These feelings of self-blame often intensified feeding challenges, particularly when premature birth had already disrupted their sense of preparedness and control.

Across several studies, mothers spoke of breastfeeding as deeply tied to cultural and societal expectations of good motherhood. Breastfeeding was often described as a responsibility and a way for mothers to demonstrate their adequacy, especially after the disruption of preterm birth [41, 44, 45, 49, 51, 53, 55]. The perceived benefits of breastmilk - optimal nutrition, growth, and protection from infections and allergies - further reinforced this sense of moral significance [41, 45, 49, 50, 52, 56]. Breastfeeding helped to counter self-blame through alignment with the ideals of good motherhood.

However, this moral framing was not universal. In one study [41], some mothers exercised agency by choosing formula feeding without experiencing the same guilt and self-blame. They believed their breastmilk lacked the nutrients their premature infant needed and that choosing formula feeding was the right thing to do. These accounts highlight that the moral meaning of breastfeeding is highly individual and shaped by prior experience, beliefs and interpretation of their infants’ needs.

These accounts show how maternal identity and agency was continually negotiated through the interplay between feeding expectations, intentions, and interpretation of the infants’ needs. As mothers worked to establish a sense of themselves as capable caregivers, their interpretations of their infant’s cues became a central part of this negotiation and are explored further in the following subtheme.

### Interpreting the infant: Making sense of cues and participation

Mothers’ interpretation of infant participation in feeding played a central role in shaping their sense of partnership in the feeding relationship. Mothers often described their infants as exerting passive and active influences on feeding. Passive influences were attributed to infants’ immaturity or physiological limitations, including difficulties coordinating sucking, swallowing and breathing; difficulties latching; weak or inconsistent sucking [38–45, 50, 51, 54, 56, 59]; fatigue or sleepiness [39, 42–44, 46, 48–50, 54, 57, 59]; and subtle or absent feeding cues [38, 42, 59]. In contrast, some mothers viewed their infants as active, intentional participants whose preferences and behaviours shaped the method and rhythm of feeding, describing infants getting excited to feed, opening their mouth to receive food and mastering their latch and suck-swallow-breathe coordination [38, 41, 59]. Some infants were seen to make an active choice about the timing and method of feeding, for instance, refusing to breastfeed and only accepting bottle feeding [48, 51]. They attributed feeding outcomes, whether successful or unsuccessful, to intentional acts by infants.

Mothers’ ability to recognise and interpret physiological and behavioural cues, depended on their awareness of preterm-related feeding difficulties [58, 59], their development of knowledge and skills [42, 49], and the clarity of feeding cues [38, 42, 59]. Misinterpreting cues combined with anxiety about adequate nutrition often led mothers to override the infant’s signals, adopting more controlling and forceful feeding practices [38, 42, 43, 45, 50, 54, 59]. Correctly interpreting cues led to reciprocal interaction and mothers feeling a sense of partnership, teamwork and understanding [49, 50, 53]. These perceptions of reciprocity shaped mothers’ confidence and how they interpreted their infants’ feeding behaviour. Ultimately it was these interpretations rather than the behaviours itself that defined feeding success or failure.

### Reframing feeding decisions: Negotiating expectations

Over time, many mothers navigated a complex process of renegotiating what it meant to feed their premature infant well, gradually reframing expectations, emotions, and their maternal identity. Providing expressed breastmilk helped some mothers maintain their perceived maternal responsibility [41, 50], and for some this led to successful transition to direct breastfeeding [50, 52, 56]. Some chose to continue to feed expressed breastmilk to have more control over the volume of milk consumed by the infant [41, 51]. Others, however, eventually shifted to formula feeding, often after months of effort, exhaustion, or concerns about their own wellbeing [39, 40, 50, 52–54]. Mothers still developed a bond with their infants even when not breastfeeding [46, 52]. A gradual shift in how mothers understood “successful feeding” marked an important turning point in their feeding journey.

Reaching this point of acceptance or re-evaluation was often a significant moment in the feeding journey. One mother, after ceasing breastfeeding, reflected on the journey towards this moment: *“Breastfeeding is not free; it is the most costly thing I have experienced. I lost myself completely”* [50]. Reframing feeding expectations, for instance deciding to use formula or stop expressing breastmilk, brought a sense of relief for some [40, 52–54], while the transition was accompanied by sadness, guilt, shame and disappointment for others [39, 41, 50–52]. These emotional responses shaped how confidently mothers could express their preferences and move forward in their feeding decisions.

Support from partners, family and friends played an important role in how mothers navigated the reframing process. Emotional encouragement, practical help, and reassurance strengthened mothers’ belief in their ability to feed and care for their infants [40–42, 45, 47, 49, 52–55]. Feeling validated strengthened mothers’ confidence and expanded their sense of choice in how to best feed their infants [38, 47, 51, 54, 57]. Conversely, lack of support made mothers question their decisions and lowered their confidence [58].

As mothers renegotiated their expectations, their confidence to voice preferences and make feeding-related decisions became central to their sense of agency [44, 50, 58]. Mothers who felt uncertain about their skills and knowledge or lacked confidence felt unheard, uncared for and as if they did not have a choice [44]. Confidence and agency further influenced help-seeking behaviour, mothers who doubted their ability to recognise feeding difficulties were less likely to seek support, often minimising concerns or feeling unsure whether they are significant enough to warrant attention [42, 47, 48, 59]. Conversely, more confident mothers actively sought solutions, either by consulting healthcare professionals or by self-testing strategies [51].

This theme highlights how mothers’ internal meaning-making and their interactions with their infants shaped their feeding experiences. Feeding a premature infant was experienced not only as a practical task but also as a moral and embodied experience shaped by labour, sacrifice, mothers’ interpretation of infant’s participation in the process and support from partners, family and friends. These individual meaning-making processes were also shaped by the wider institutional and societal systems surrounding them, dynamics explored in the themes that follow.

### Navigating the institutional landscape

While mothers’ sense of agency was shaped by their own meaning-making, interactions with their infants, and support from existing networks, it was also influenced by the institutional structures surrounding them. Although healthcare policies and resources vary widely across countries and between rural and urban settings, the institutional influence on feeding appears remarkably consistent, with mothers across diverse contexts describing similar challenges relating to hospital routines, professional expectations, power dynamics, and the organisational culture of neonatal and community care. This theme explores how these challenges and experiences shaped the feeding journey and continued to influence mothers’ efforts long after they left neonatal care.

### Institutional routines and their lasting imprint on feeding

The highly structured routines and practices in hospital environments left lasting imprints on feeding practices and maternal agency. The emphasis on weight gain as a discharge criterion, combined with institutional policies promoting breastfeeding in many countries, created a complex and often conflicting landscape for mothers. Weight became a visible measure of feeding success, yet breastfeeding was framed as the morally superior option. Navigating these dual expectations left mothers feeling torn, distressed and uncertain about how to meet both standards [44, 47, 49]. Some mothers described being urged to bottle feed to track intake, whilst others described the frustration of being encouraged to start breastfeeding when the infant was not ready [44]. These dual expectations shaped not only feeding practices in the hospital but also the confidence and decision-making mothers carried into feeding at home.

The hospital stay shaped infants’ feeding behaviours and mothers’ sense of agency, confidence and choice in feeding, with many mothers describing a loss of control over their feeding goals and pressure to conform to guidelines, such as offering expressed breastmilk by bottle to hasten discharge [41, 47, 49, 51]. This pressure made it harder for mothers to attune themselves to their infant’s cues and experience feeding as enjoyable and relational [47]. Institutional emphasis on weight gain frequently made feeding task-oriented, reducing reciprocity and diminishing maternal agency [38, 49, 54]. Hospital practices became either a source of confidence or constraint influencing feeding at home.

Hospital guidance shaped feeding well beyond discharge, with skills learned in hospital providing confidence for some [38, 42], and practices such as early bottle feeding and limiting breastfeeding leading others to have ongoing challenges including infant refusal to breastfeed and bottle preference [41, 47, 51, 52, 55]. These experiences show how institutional pressures can follow families home, shaping how mothers perceive and respond to support after discharge.

### Professional guidance and support

Taking a premature infant home placed mothers under intense pressure, and professional reassurance, particularly guidance that feeding difficulties are common and that feeding should work for the mother, infant, and family helped them to renegotiate feeding expectations and build confidence that were otherwise undermined by inconsistent or absent advice [41, 45, 47, 53].

Many mothers described significant gaps in preparation for discharge and limited prematurity-specific expertise within community services leaving them with unanswered questions about breastfeeding techniques, conflicting professional advice and the need to seek information themselves [39, 40, 42–45, 51–55]. Mothers relied on their own persistence, often turning to friends, family, other parents of premature infants, or online resources for help [39, 51, 55]. This lack of prematurity-specific advice coupled with intense scrutiny and surveillance after discharge, left some mothers feeling the burden of responsibility without the tools and support needed to achieve feeding goals [47].

The transition home marked a critical juncture where the presence or absence of institutional support shaped mothers’ emerging agency and confidence. Intense surveillance, inadequate support and contradictory information shaped mothers’ feeding experiences, emotional wellbeing and their sense of responsibility for meeting externally defined standards. These institutional pressures were further reinforced by wider societal expectations explored in the next theme.

#### Impact of societal norms on feeding experiences

Across many countries, public health messaging constructs breastfeeding as the optimal and morally preferred choice, creating a powerful backdrop that leaves mothers of premature infants vulnerable to judgement when their realities diverge from these ideals. Mothers found that public health messages failed to account for the realities of prematurity, leaving their feeding decisions open to scrutiny from family, friends, professionals, and the wider public and intensifying the sense of cultural pressure and surveillance [49, 51].

Critical ideals of ‘good motherhood’ were reinforced through social media, advertising and public health campaigns idealising the nutritional and relational benefits of breastfeeding [49]. A sense of social surveillance and stigma left mothers feeling pressured to conform to breastfeeding norms and ashamed when they could not, with some describing embarrassment when buying formula or bottle feeding in public [49, 51, 52]. Some mothers altered their feeding practices to avoid judgement, even when this conflicted with infant cues or feeding preferences, such as pretending to breastfeed in public even when the infant is actually bottle-fed [49, 51, 52], leading to some mothers avoiding feeding in public and eroded their confidence and agency [59]. Such shame and self-blame reflect a pervasive societal narrative that positions mothers as being primarily responsible for the success of infant feeding.

Societal expectations varied markedly by country, unlike institutional and interpersonal influences, which were relatively consistent across contexts. In Scandinavian settings with strong pro-breastfeeding norms, mothers described shame and stigma when bottle-feeding in public [49, 51, 52], whereas in other cultural contexts, mothers reported embarrassment when breastfeeding in public [59]. These cultural differences reveal that societal norms are not universal but socially constructed, shaping mothers’ feeding experiences in profoundly different ways.

### Integrating the findings: Navigating the feeding journey

Feeding decisions are not a single moment but rather an evolving negotiation across the infant’s first year. Mothers often wrestle with the gap between their feeding intentions and the realities of feeding a premature infant at home, a journey many feel unprepared for. The struggle to build and sustain maternal agency and confidence does not end at discharge; for many, it intensifies at home. Although not under the direct gaze of healthcare professionals, institutional and societal expectations continue to shape mothers’ sense of what is ‘right’. This influences mothers’ confidence and ability to make feeding decisions that align with their infant’s needs, their own wellbeing, and family life.

**Fig. 2:**
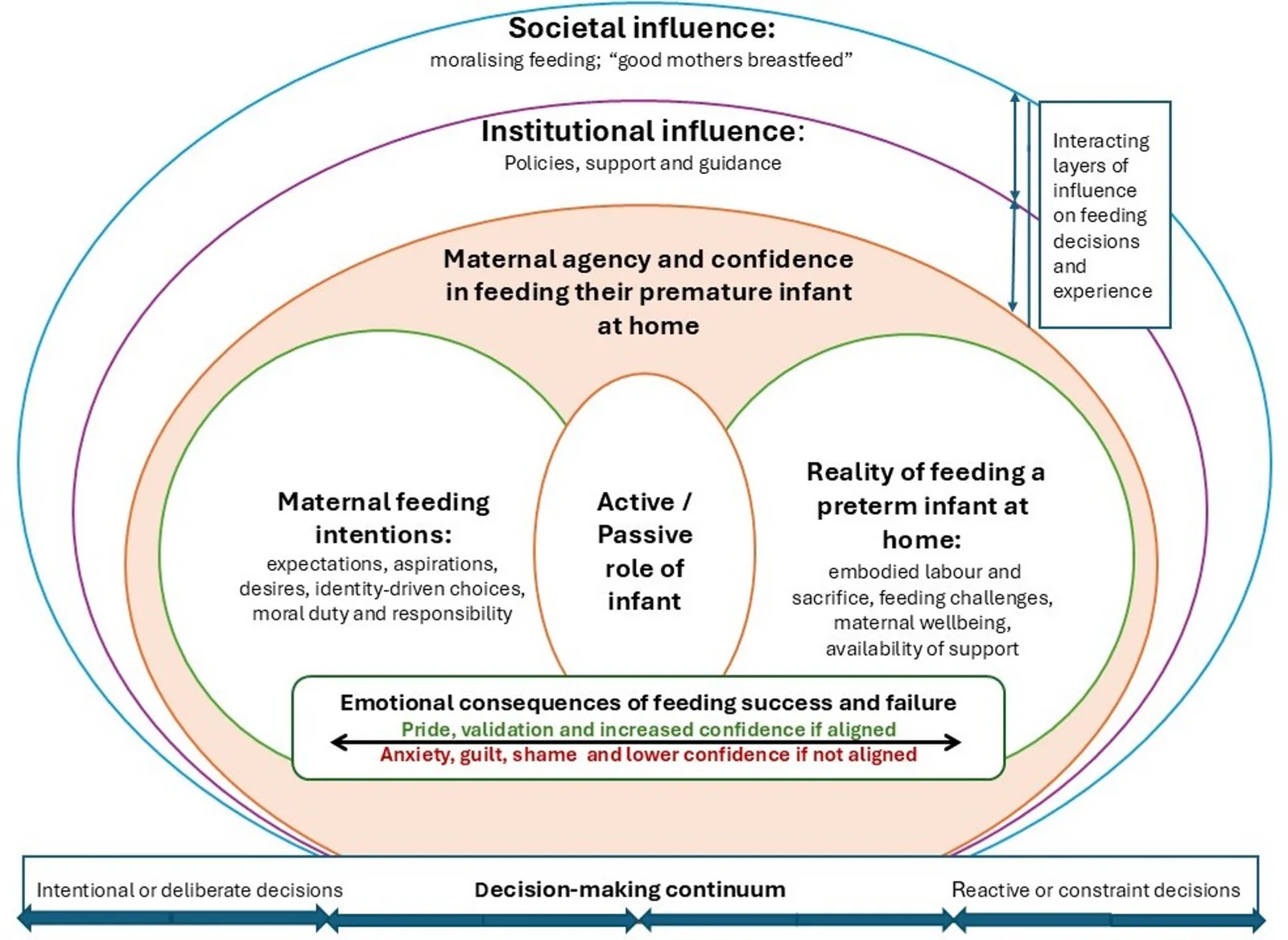
Navigating the feeding journey

We developed a conceptual model to capture how these influences interact over time (Fig. 2). Drawing on the socioecological perspective, the model integrates the findings and illustrates how mothers navigate the complex and shifting landscape of feeding a premature infant at home through interacting layers of influence. During this journey mothers oscillate between intentional and deliberate or reactive and constrained feeding decisions, while developing their agency and confidence in feeding their premature infant.

The inner circle of the model emphasises the emotional turmoil of giving birth early and the tension it creates between intentions and the reality of feeding a premature infant. Infant behaviours shape the feeding experience, whether passive prematurity-related behaviour (such as sleepiness or coordination difficulties) or cues that mothers interpret as active participation or avoidance. These interactions alongside incremental mastery of feeding skills, embodied sacrifice and the emotional consequences of perceived feeding success and failure often lead to the reframing of feeding expectations and continue to impact on maternal agency and confidence.

Furthermore, the quality of support available to mothers is essential to the development of maternal agency and confidence. Navigating institutional and societal expectations, including conflicting messages about breastfeeding, growth, and ‘good motherhood’, continues to shape mothers’ decisions long after discharge. The decision-making continuum illustrates how mothers’ feeding decisions shifted over time between more intentional/deliberate and more reactive/constrained responses, depending on their confidence, contextual pressures and infant feeding behaviours.

This model highlights that supporting mothers of premature infants requires more than addressing feeding techniques. It involves strengthening the conditions that enable mothers to build and sustain agency over time. Supporting mothers to interpret their infants’ cues, navigate institutional expectations, and resist unhelpful societal pressures, position them to make the best feeding decisions for them and their infant and to participate confidently in shared decision-making in future healthcare encounters. By recognising the influences that shape the feeding journey, practitioners and researchers can design interventions that not only meet immediate feeding needs but also foster the relational, emotional, and structural foundations that empower mothers to engage as equal partners in their child’s ongoing care.

## Discussion

This systematic review and thematic synthesis explored mothers’ experiences of feeding their premature infants in the early months at home. Across diverse countries and care systems, mothers describe remarkably similar emotional, practical, and informational challenges. Parental experiences after discharge have remained largely unchanged over the last 25 years despite advances in neonatal care. Feeding a premature infant emerged not as a technical task but a complex, relational, identity-laden process influenced by multiple layers of the socio-ecological system surrounding the mother.

A central finding was that many mothers of premature infants must fundamentally reframe what it means to feed their infants well. Feeding was often interpreted as a moral and embodied experience with perceived success or failure directly influencing maternal agency. Consistent with previous research, breastfeeding difficulties were often interpreted as moral failures, leading to feelings of guilt, shame and stigma [65, 66]. These interpretations often led mothers to internalise feeding challenges not as developmental characteristics of prematurity but as personal shortcomings. This synthesis highlights that reassurance and guidance from healthcare professionals can help reduce the emotional burden, yet many mothers reported receiving inconsistent or conflicting advice.

Maternal agency was also shaped by the degree of reciprocity and attunement mothers establish with their infants. The meaning mothers assign to their premature infants’ feeding cues, whether passive signs of immaturity or active participation, strongly influenced mothers’ confidence and the emotional tone of feeding interactions. Cue-based feeding is recognised as best practice [67, 68], yet developing mind-mindedness after discharge is complicated by subtle cues, heightened anxiety, and limited feeding knowledge [5, 6, 69].

This review extends prior work by showing that feeding a premature infant is a skill that must be learned and refined after discharge. Each feeding experience offers an opportunity for mothers to move closer to or further away from mind-mindedness and attunement. When mothers correctly interpret their infant’s cues, reciprocity increases, and feeding becomes a more joyful, connected experience; when cues are missed or overridden, often due to anxiety about weight gain, reciprocity diminishes [5, 6, 69], reinforcing or eroding agency in the process.

Institutional protocols, professional expectations, and societal pressures further shape maternal agency. Consistent with findings of previous studies, this synthesis revealed that practices such as restricting direct breastfeeding, test weighting (weighing the infant before and after feeding) and emphasis on weight gain as a criteria for discharge sit in tension with breastfeeding promotion and broader societal pressure to breastfeed [70]. Some mothers found these practices stressful, increasing the risk of coercive feeding practices [5, 7, 71, 72], while others experienced them as reassuring [73]. Regardless of interpretation, institutional influences extend well beyond discharge, shaping maternal confidence, decision-making, and feeding relationships at home. This underscores the need for individualised family-centred care that recognises and responds to the unique strengths, needs, and pressures within each mother–infant dyad.

Support from healthcare professionals was highly valued when consistent and responsive [74, 75]. However, consistent with other studies, this synthesis found maternal dissatisfaction with feeding support during discharge and the early months at home [12, 23]. Conflicting messages between professionals, and between professional advice and mothers’ own feeding decisions and experiences, often disrupt maternal agency and undermines trust in their ability to read their infant’s cues [7, 74, 76].

Societal expectations also played a role. Norms of ‘good feeding’ and ‘good motherhood’ generated feelings of shame and embarrassment for mothers across the spectrum [65]. For example, some mothers felt embarrassed about breastfeeding in public, whereas others felt embarrassed about bottle feeding. In an increasingly migratory world, many mothers must reconcile the norms of their culture of origin with those of their host country, creating additional layers of tension and uncertainty that can further complicate feeding decisions and undermine maternal agency. These contradictions illustrate how societal norms can undermine maternal confidence not only through their content but also through their inconsistency, leaving mothers to be judged regardless of their feeding choices. However, it is important to note that much of the existing research excludes mothers who do not speak the official language of the research country, meaning that the societal norms shaping their feeding experiences and their unique pressures remain largely undocumented [77, 78].

Although many studies and reviews have examined individual elements of the socio-ecological model, few have considered how these layers interact to shape feeding experiences of mothers of premature infants after discharge. This review brings these influencing layers together, offering a more integrated lens through which to understand the complex reality parents face when feeding their premature infants at home. This synthesis of evidence across relational, institutional, and societal domains highlights how multiple systems converge to influence maternal agency, feeding confidence, and ultimately feeding outcomes.

### Limitations

This qualitative systematic review and thematic synthesis have several limitations. Only studies published in English or those for which translation was available through the host website were included. Although the consistency of findings across countries and over time suggests that additional non-English studies may not have altered the core themes, their exclusion may mean that some culturally specific experiences remain under-represented. Furthermore, the included studies predominately came from high-income countries with well-established neonatal care systems, only limited represented middle-income countries and none from low-income countries.

Full dual screening is not typically required for thematic synthesis, 30–35% of records were dual-screened in line with our a priori protocol, which may have introduced some potential for missed records.

Although not an explicit inclusion criterion, all included studies focused exclusively on mothers’ experiences of feeding their premature infants. This inevitably limits the perspectives captured, by excluding those of fathers, partners, and wider family members, who play important roles in feeding and decision-making.

Finally, the included studies collected data at various points within the first year after discharge. Mothers often described their feeding journey as a continuous experience rather than as distinguishing between hospital and home, some reflections may therefore relate to their inpatient stay. While this limits the precision of post-discharge attribution, it more accurately reflects how parents experience feeding as an ongoing, evolving process.

### Clinical and research implications

There is a need for an institutional shift from viewing feeding as an isolated mechanical and physiological activity with the sole purpose of achieving weight gain and growth. The findings of this review demonstrate that supporting families after discharge cannot rely solely on technical expertise or weight-focused monitoring but must also address the emotional labour, identity work, and social pressures that shape maternal agency. Strengthening continuity between neonatal and community services, ensuring consistent and culturally attuned guidance, and recognising the diverse realities of families are essential steps toward closing the gap between clinical survival and lived experience. Changing the focus of care will not only create conditions in which parents can develop agency and confidence in feeding but also engage in decision-making without feeling directed, judged, or sidelined.

This review highlights the need for a deeper exploration of the feeding experiences of minority, marginalised and linguistically diverse communities who are frequently excluded from research. Understanding parents’ experiences of feeding their premature infants after discharge and the layers of the socio-ecological model that shape each parents’ experience is essential for developing support and interventions that acknowledge the complexity of the journey, inform practice and prepare the workforce to meet the needs of all families.

## Conclusions

Parental experiences of feeding premature infants at home have remained remarkably consistent in their core biological and psychological challenges since 2000, despite considerable advances in medical technology, survival rates and the widespread shift toward viewing feeding as a relational process rather than a mechanical task. Although parents today have access to more frequent, person-centred, and technology-enhanced support, the fundamental difficulties of feeding a premature infant at home persist. Ultimately, supporting parents of premature infants requires attention not only to feeding techniques but also to the relational, institutional, and societal forces that shape maternal agency and confidence long after hospital discharge.

## Supplementary Information

Below is the link to the electronic supplementary material.

Supplementary Material 1

Supplementary Material 2

## Data Availability

The datasets supporting the conclusions of this article are included within the article and its additional files.

## Abbreviations

GRADE-CERQual: Confidence in the Evidence from Reviews of Qualitative research
NICU: neonatal intensive care unit
