# Supplementary material for: Mothers’ experiences of feeding their premature infants after discharge from the hospital: a systematic review and thematic synthesis
Source: Int Breastfeed J. 2026 Sep 9;21:91. doi: 10.1186/s13006-026-00893-y (PMC13637324; doi:10.1186/s13006-026-00893-y)
Supplement: Supplementary file 1 [file 13006_2026_893_MOESM1_ESM.docx]

**Mothers’ experiences of feeding their premature infants after discharge from the hospital: a systematic review and thematic synthesis**

## Evidence search report

## Completed: 12th March, 2025

## Search strategy

**Ovid - Medline**

**1.** exp Infant, Premature/ or Infant, Low Birth Weight/

**2.** (premie* or preemie* or preterm or pre term or premature or low birth weight).mp.

**3.** 1 or 2

**4.** Parents/ or Mothers/ or Fathers/ or Caregivers/ or exp Parent-Child Relations/ or Caregiver Burden/ or exp Emotions/ or Psychosocial Functioning/

**5.** (parent* or mother* or father* or caregiver* or guardian* or parent* attune* or parent-child relation* or mother-child relation* or father-child relation* or caregiver burden* or self-efficacy or psychosocial function* or challenge* or hope* or anxiet* or emotion*).mp.

**6.** 4 or 5

**7.** Feeding Behavior/ or Bottle Feeding/ or Breast Feeding/ or Sucking Behavior/ or Eating/ or Drinking/ or Weaning/ or Food Fusiness/ or Food Preferences/

**8.** (infant* feed* or feed* behavi*r or bottle feed* or bottle fed or breast feed* or breastfeed* or breastfed or suck* behavi*r or food fussiness or fuss* or food preference* or eat* or drink* or wean* or cue-based or cue* or complementary or infant-driven).mp.

**9.** 7 or 8

**10.** "Continuity of Patient Care"/ or Aftercare/ or Hospital to Home Transition/ or Patient Discharge/ or Patient Transfer/ or Transitional Care/

**11.** (care contin* or aftercare or hospital-to-home transition* or home or patient discharge or patient transfer or transitional care or after discharge or post-discharge or discharge or follow-up or community or transition* or post-hospitali*ation or posthospitali*ation).mp.

**12.** 10 or 11

**13.** 3 and 6 and 9 and 12

**14.** limit 13 to yr="2000 -Current"

**Ovid - Embase**

**1.** exp prematurity/ or low birth weight/

**2.** (premie* or preemie* or preterm infant* or pre term infant* or premature infant* or low birth weight).mp.

**3.** 1 or 2

**4.** parent/ or mother/ or father/ or caregiver/ or exp child parent relation/ or caregiver burden/ or exp emotion/

**5.** (parent* or mother* or father* or caregiver* or guardian* or parent* attune* or parent-child relation* or mother-child relation* or father-child relation* or caregiver burden* or self-efficacy or psychosocial function* or challenge* or hope* or anxiet* or emotion*).mp.

**6.** 4 or 5

**7.** feeding behavior/ or exp infant feeding/ or sucking/ or eating/ or drinking/ or weaning/ or food fussiness/ or food preference/

**8.** (infant* feed* or feed* behavi*r or bottle feed* or bottle fed or breast feed* or breastfeed* or breastfed or suck* behavi*r or food fussiness or fuss* or food preference* or eat* or drink* or wean* or cue-based or cue* or complementary or infant-driven).mp.

**9.** 7 or 8

**10.** exp aftercare/ or exp hospital discharge/ or transitional care/

**11.** (care contin* or aftercare or hospital-to-home transition* or home or patient discharge or patient transfer or transitional care or after discharge or post-discharge or discharge or follow-up or community or transition* or post-hospitali*ation or posthospitali*ation).mp.

**12.** 10 or 11

**13.** 3 and 6 and 9 and 12

**14.** limit 13 to yr="2000 -Current"

**Ebsco - CINAHL**

**S14** S3 AND S6 AND S9 AND S12 Limiters - Publication Date: 20000101-20251231

**S13** S3 AND S6 AND S9 AND S12

**S12** S10 OR S11

**S11** care contin* or aftercare or hospital-to-home transition* or home or patient discharge or patient transfer or transitional care or after discharge or post-discharge or discharge or follow-up or community or transition* or post-hospitali*ation or posthospitali*ation

**S10** (MH "Continuity of Patient Care+") OR (MH "After Care") OR (MH "Patient Discharge") OR (MH "Transfer, Discharge") OR (MH "Transitional Care")

**S9** S7 OR S8

**S8** infant* feed* or feed* behavi*r or bottle feed* or bottle fed or breast feed* or breastfeed* or breastfed or suck* behavi*r or food fussiness or fuss* or food preference* or eat* or drink* or wean* or cue-based or cue* or complementary or infant-driven

**S7** (MH "Infant Feeding") OR (MH "Breast Feeding") OR (MH "Bottle Feeding") OR (MH "Sucking Behavior") OR (MH "Eating") OR (MH "Infant Weaning") OR (MH "Food Fussiness") OR (MH "Food Preferences")

**S6** S4 OR S5

**S5** parent* or mother* or father* or caregiver* or guardian* or parent* attune* or parent-child relation* or mother-child relation* or father-child relation* or caregiver burden* or self-efficacy or psychosocial function* or challenge* or hope* or anxiet* or emotion*

**S4** (MH "Parents") OR (MH "Fathers") OR (MH "Mothers") OR (MH "Caregivers") OR (MH "Parent-Child Relations+") OR (MH "Parent-Infant Relations+") OR (MH "Caregiver Burden") OR (MH "Emotions+") OR (MH "Psychosocial Functioning")

**S3** S1 OR S2

**S2** premie* or preemie* or preterm or pre term or premature or low birth weight

**S1** (MH "Infant, Premature") OR (MH "Infant, Low Birth Weight")

**Proquest/Dialog - PsycInfo**

**S1** ti(premie* or preemie* or "preterm infant*" or "pre term infant*" or "premature infant*" or preterm or "pre term" or premature or "low birth weight") OR ab(premie* or preemie* or "preterm infant*" or "pre term infant*" or "premature infant*" or preterm or "pre term" or premature or "low birth weight")

**S2** (SU.EXACT("Parents" OR "Stepparents" OR "Foster Parents" or "Homosexual Parents" OR "Single Parents" OR "Adoptive Parents" OR "Caregivers" OR "Parental Mental Health" OR "Parental Stress" OR "Caregiver Burden") OR SU.EXACT.EXPLODE("Fathers" OR "Mothers" OR "Parent Child Relations" OR "Emotional States"))

**S3** (ti(parent* or mother* or father* or caregiver* or guardian* or "parent* attune*" or "parent-child relation*" or "mother-child relation*" or "father-child relation*" or "caregiver burden*" or self-efficacy or "psychosocial function*" or challenge* or hope* or anxiet* or emotion*) OR ab(parent* or mother* or father* or caregiver* or guardian* or "parent* attune*" or "parent-child relation*" or "mother-child relation*" or "father-child relation*" or "caregiver burden*" or self-efficacy or "psychosocial function*" or challenge* or hope* or anxiet* or emotion*))

**S4** S3 OR S2

**S5** (SU.EXACT("Bottle Feeding" OR "Weaning" OR "Breast Feeding" OR "Eating Behavior" OR "Sucking" OR "Drinking Behavior" OR "Food Preferences"))

**S6** (ti("infant* feed*" or "feed* behavi*r" or "bottle feed*" or "bottle fed" or "breast feed*" or breastfeed* or breastfed or "suck* behavi*r" or "food fussiness" or fuss* or "food preference*" or eat* or drink* or wean* or cue-based or cue* or complementary or infant-driven) OR ab("infant* feed*" or "feed* behavi*r" or "bottle feed*" or "bottle fed" or "breast feed*" or breastfeed* or breastfed or "suck* behavi*r" or "food fussiness" or fuss* or "food preference*" or eat* or drink* or wean* or cue-based or cue* or complementary or infant-driven))

**S7** S6 OR S5

**S8** (SU.EXACT("Continuum of Care" OR "Hospital Discharge" OR "Aftercare" OR "Discharge Planning"))

**S9** (ti("care contin*" or aftercare or "hospital-to-home transition*" or home or "patient discharge" or "patient transfer" or "transitional care" or "after discharge" or post-discharge or discharge or follow-up or community or transition* or post-hospitali*ation or posthospitali*ation) OR ab("care contin*" or aftercare or "hospital-to-home transition*" or home or "patient discharge" or "patient transfer" or "transitional care" or "after discharge" or post-discharge or discharge or follow-up or community or transition* or post-hospitali*ation or posthospitali*ation))

**S10** S9 OR S8

**S11** S10 AND S7 AND S4 AND S1

**Proquest/Dialog - AMED**

**S1** (SU.EXACT("INFANT PREMATURE" OR "INFANT LOW BIRTH WEIGHT"))

**S2** ti(premie* or preemie* or preterm or "pre term" or premature or "low birth weight") OR ab(premie* or preemie* or preterm or "pre term" or premature or "low birth weight")

**S3** S2 OR S1

**S4** (SU.EXACT.EXPLODE("PARENTS" OR "PARENT CHILD RELATIONS" OR "EMOTIONS") OR SU.EXACT("CAREGIVERS"))

**S5** ti(parent* or mother* or father* or caregiver* or guardian* or "parent* attune*" or "parent-child relation*" or "mother-child relation*" or "father-child relation*" or "caregiver burden*" or self-efficacy or "psychosocial function*" or challenge* or hope* or anxiet* or emotion*) OR ab(parent* or mother* or father* or caregiver* or guardian* or "parent* attune*" or "parent-child relation*" or "mother-child relation*" or "father-child relation*" or "caregiver burden*" or self-efficacy or "psychosocial function*" or challenge* or hope* or anxiet* or emotion*)

**S6** S5 OR S4

**S7** (SU.EXACT("FEEDING BEHAVIOR" OR "BREAST FEEDING" OR "EATING"))

**S8** ti("infant* feed*" or "feed* behavi*r" or "bottle feed*" or "bottle fed" or "breast feed*" or breastfeed* or breastfed or "suck* behavi*r" or "food fussiness" or fuss* or "food preference*" or eat* or drink* or wean* or cue-based or cue* or complementary or infant-driven) OR ab("infant* feed*" or "feed* behavi*r" or "bottle feed*" or "bottle fed" or "breast feed*" or breastfeed* or breastfed or "suck* behavi*r" or "food fussiness" or fuss* or "food preference*" or eat* or drink* or wean* or cue-based or cue* or complementary or infant-driven)

**S9** S8 OR S7

**S10** (SU.EXACT("CONTINUITY OF PATIENT CARE" OR "AFTER CARE" OR "PATIENT DISCHARGE" OR "PATIENT TRANSFER"))

**S11** ti("care contin*" or aftercare or "hospital-to-home transition*" or home or "patient discharge" or "patient transfer" or "transitional care" or "after discharge" or post-discharge or discharge or follow-up or community or transition* or post-hospitali*ation or posthospitali*ation) OR ab("care contin*" or aftercare or "hospital-to-home transition*" or home or "patient discharge" or "patient transfer" or "transitional care" or "after discharge" or post-discharge or discharge or follow-up or community or transition* or post-hospitali*ation or posthospitali*ation)

**S12** S11 OR S10

**S13** S12 AND S9 AND S6 AND S3
