# Supplementary material for: Mothers’ experiences of feeding their premature infants after discharge from the hospital: a systematic review and thematic synthesis
Source: Int Breastfeed J. 2026 Sep 9;21:91. doi: 10.1186/s13006-026-00893-y (PMC13637324; doi:10.1186/s13006-026-00893-y)
Supplement: Supplementary file 2 [file 13006_2026_893_MOESM2_ESM.docx]

**Mothers’ experiences of feeding their premature infants after discharge from the hospital: a systematic review and thematic synthesis**

**Supplementary information**

**GRADE -CERQual Summary of Qualitative Findings Tables**

**Finding 1: Feeding a preterm infant in the early weeks is physically and emotionally demanding, shaped by intensive routines, exhaustion, and moments of connection.**

| Component | Assessment | Explanation |
| --- | --- | --- |
| Studies contributing to this finding | (1–21) | 21/22 studies contributed to this finding |
| Methodological limitations | Minor concerns | 11 scored high, 8 moderate and 2 low on CASP for methodological quality, main issue was insufficient description of participant recruitment and data collection and lack of detail about reflexivity. The two studies with low quality did not contribute significantly to the finding. |
| Coherence | Minor concerns | This finding is broadly supported by the data. More studies presented data about the physical and emotional demand of feeding, fewer mentioned the joy and connective moments with the infant. |
| Adequacy of data | Minor concerns | Using the Richness and Thickness Assessment Tool, 17/21 studies had moderate to large amount of data relating to this finding. Adequately thick contextual descriptions were present for 9/21 and rich conceptual data for 12/21 studies. These higher quality and richer studies contributed most to this finding, supported by data from the other studies. |
| Relevance | Minor concerns | Included studies were conducted between 2000 and 2024 and in a range of countries with different healthcare systems. Topic of interest: 9/21 had direct relevance (8 breastfeeding experiences, 1 all feeding); 3/21 partial relevance (2 breastfeeding and 1 general feeding decision making); 8/21 indirect relevance (2 general caring for preterm, 4 experiences of community support, 3 theoretical development). |
| Overall CERQual assessment | **High confidence** | Supported by rich, consistent evidence with only minor concerns. |

**Finding 2: Breastfeeding is not simply a nutritional act or a technical skill but a deeply symbolic and relational practice through which mothers negotiated identity, responsibility, and the emotional aftermath of premature birth.**

| Component | Assessment | Explanation |
| --- | --- | --- |
| Studies contributing to this finding | (1–21) | 21/22 studies contributed to this finding |
| Methodological limitations | Minor concerns | 11 scored high, 8 moderate and 2 low on CASP for methodological quality, main issue was insufficient description of participant recruitment and data collection and lack of detail about reflexivity. The two studies with low quality did not contribute significantly to the finding. |
| Coherence | Minor concerns | This finding is broadly supported by the data. Mothers consistently describe fluctuating agency, though the concept was interpreted differently across studies. Highly consistent descriptions of breastfeeding as tied to maternal identity. |
| Adequacy of data | Minor concerns | Using the Richness and Thickness Assessment Tool, 18/21 studies had moderate to large amount of data relating to this finding. Adequately thick contextual descriptions were present for 10/19 and rich conceptual data for 13/19 studies. These studies contributed most to the finding, with 8 of these studies conducted in Scandinavian countries. The finding was however supported by studies from other parts of the world. |
| Relevance | Minor concerns | Included studies were conducted between 2000 and 2024 and in a range of countries with different healthcare systems. Topic of interest: 10/21 had direct relevance (9 breastfeeding experiences, 1 all feeding); 3/21 partial relevance (2 breastfeeding and 1 general feeding decision making); 8/19 indirect relevance (2 general caring for preterm, 4 experiences of community support, 2 theoretical development). |
| Overall CERQual assessment | **High confidence** | Supported by rich, consistent evidence with only minor concerns about coherence, specifically about different descriptions and interpretations of identity. |

**Finding 3: The meaning mothers assign to infant cues played a central role in shaping maternal agency, confidence, and the emotional tone of the feeding relationship.**

| Component | Assessment | Explanation |
| --- | --- | --- |
| Studies contributing to this finding | (1,2,4–7,9–19,21) | 19/22 studies contributed to this finding |
| Methodological limitations | Minor concerns | 9 scored high, 7 moderate and 3 low on CASP for methodological quality, main issue was insufficient description of participant recruitment and data collection and lack of detail about reflexivity. The three studies with low quality did not contribute significantly to the finding but supported data from other studies. |
| Coherence | Moderate concerns | Strong consistency in descriptions of infant‑driven influences. Not all studies explicitly linked the infant’s participation with maternal agency although the impact on the mother was visible in many of the quotes. |
| Adequacy of data | Minor concerns | Using the Richness and Thickness Assessment Tool, 12/19 studies had moderate to large amount of data relating to this finding. Adequately thick contextual descriptions were present for 8/19 and rich conceptual data for 11/19 studies. These higher quality and richer studies contributed most to this finding, supported by data from the other studies. |
| Relevance | Minor concerns | Included studies were conducted between 2000 and 2024 and in a range of countries with different healthcare systems. Topic of interest: 10/19 had direct relevance (9 breastfeeding experiences, 1 all feeding); 3/19 partial relevance (2 breastfeeding and 1 general feeding decision making); 6/19 indirect relevance (2 general caring for preterm, 2 experiences of community support, 2 theoretical development). Finding highly relevant across gestational ages and contexts. |
| Overall CERQual assessment | **Moderate confidence** | Moderate concerns about coherence, specifically about different descriptions and interpretations of agency and confidence reduce confidence slightly. |

**Finding 4: Reframing feeding decisions involve redefining what it meant to be a ‘good mother’, shifting the focus from the method of feeding to the infant’s health, growth, the feeding relationship and the mothers’ own wellbeing.**

| Component | Assessment | Explanation |
| --- | --- | --- |
| Studies contributing to this finding | (1,3,5,6,9–13,17–21) | 14/22 studies contributed to this finding |
| Methodological limitations | No or very minor concerns | 10 scored high, 3 moderate and 1 low on CASP for methodological quality, main issue was insufficient detail about reflexivity for the moderate studies. The study deemed low quality had a general lack of methodological detail, this study supported other studies rather than contributing much to the finding. |
| Coherence | Minor concerns | Some variation in how mothers reframed decisions; not all studies explored this deeply. |
| Adequacy of data | Minor concerns | Using the Richness and Thickness Assessment Tool, 9/14 studies had moderate to large amount of data relating to this finding. Adequately thick contextual descriptions were present for 6/14 and rich conceptual data for 10/14 studies. Fewer studies contributed detailed accounts, and it mostly related to cessation of breastfeeding. |
| Relevance | Minor concerns | Included studies were conducted between 2000 and 2023, in a range of countries with different healthcare systems and included different levels of prematurity. Topic of interest: 8/14 had direct relevance; 6/10 indirect relevance (2 general caring for preterm, 2 experience of community support, 2 theoretical development). |
| Overall CERQual assessment | **Moderate confidence** | Moderate concerns about coherence and richness of descriptions, confidence therefore reduced. |

**Finding 5: Institutional structures and availability and adequacy of support shape not only feeding practices within the hospital but also mothers’ confidence and agency in decision-making and the feeding relationship long after discharge.**

| Component | Assessment | Explanation |
| --- | --- | --- |
| Studies contributing to this finding | (1–3,6,8–11,13–15,18–20,22) | 15/22 studies contributed to this finding |
| Methodological limitations | Minor concerns | 7 scored high, 6 moderate and 2 low on CASP for methodological quality, main issue was insufficient description of participant recruitment and data collection and lack of detail about reflexivity. The two studies with low quality did not contribute significantly to the finding. |
| Coherence | No or very minor concerns | Strongly consistent descriptions of institutional influence on maternal confidence and agency in feeding. Studies give voice to both positive and negative views of their hospital stay and support post-discharge. |
| Adequacy of data | Minor concerns | Using the Richness and Thickness Assessment Tool, 11/15 studies had moderate to large amount of data relating to this finding. Adequately thick contextual descriptions were present for 8/15 and rich conceptual data for 10/15 studies. Some studies provided limited detail on hospital practices since selected studies focused on post-discharge feeding. The higher quality and richer studies contributed most to this finding, especially relating to post-discharge support, supported by data from the other studies. |
| Relevance | No or very minor concerns | Included studies were conducted between 2000 and 2024 and in a range of countries with different healthcare systems and included different levels of prematurity. Topic of interest related to this finding: 8/15 had direct relevance (support and decision-making post-discharge); 8/15 partial relevance (breastfeeding experience); 1/15 indirect relevance (2 theoretical development). |
| Overall CERQual assessment | **High confidence** | Supported by rich, consistent evidence with only minor concerns. |

**Finding 6: Societal messages of ‘good mothering’ and good feeding often fail to acknowledge the unique challenges associated with prematurity.**

| Component | Assessment | Explanation |
| --- | --- | --- |
| Studies contributing to this finding | (6,12,16,18,20) | 5/22 studies contributed to this finding |
| Methodological limitations | No or very minor concerns | 2 scored high and 3 moderate on CASP for methodological quality, main issue was insufficient detail about reflexivity. |
| Coherence | No or very minor concerns | Consistent descriptions of stigma and surveillance. |
| Adequacy of data | Serious concerns | Using the Richness and Thickness Assessment Tool, 5/5 studies had moderate to large amount of data relating to this finding. Adequately thick contextual descriptions were present for 3/5 and rich conceptual data for 4/5 studies. Quantity of data was limited, with only 5 studies contributing to the review finding, and 4/5 of these from Scandinavian countries. |
| Relevance | Serious concerns | Included studies were conducted between 2007 and 2019, in a limited range of mainly Scandinavian countries. Different levels of prematurity included. Topic of interest: 5/5 had direct relevance. Evidence concentrated in Scandinavian contexts; limited cultural diversity. |
| Overall CERQual assessment | **Low confidence** | Concerns about adequacy and relevance reduce confidence. |

**Bibliography**

1.	Breivold K, Hjaelmhult E, Sjöström‐Strand A, Hallström IK. Mothers’ experiences after coming home from the hospital with a moderately to late preterm infant – a qualitative study. Scand J Caring Sci. 2019;33(3):632–40. doi:10.1111/scs.12656

2.	Dosani A, Hemraj J, Premji SS, Currie G, Reilly SM, Lodha AK, et al. Breastfeeding the late preterm infant: experiences of mothers and perceptions of public health nurses. Int Breastfeed J. 2017;12(1):23. doi:10.1186/s13006-017-0114-0

3.	Ericson J, Flacking R, Udo C. Mothers’ experiences of a telephone based breastfeeding support intervention after discharge from neonatal intensive care units: a mixed-method study. Int Breastfeed J. 2017;12(Journal Article):50. doi:10.1186/s13006-017-0142-9

4.	Ericson J, Palmér L. Cessation of breastfeeding in mothers of preterm infants-A mixed method study. PloS One. 2020;15(5):e0233181. doi:10.1371/journal.pone.0233181 PubMed PMID: 32413062; PubMed Central PMCID: PMC7228110.

5.	Espitia EC, Ocampo González MP. Retrieving the offspring and caring for it during the first night at home. Investig Educ En Enfermeria. 2013;31(3):354–63. doi:10.17533/udea.iee.17495

6.	Flacking R, Ewald U, Starrin B. ‘I wanted to do a good job’: experiences of ‘becoming a mother’ and breastfeeding in mothers of very preterm infants after discharge from a neonatal unit. Soc Sci Med. 6 AD;64(12):2405–16. doi:10.1016/j.socscimed.2007.03.008

7.	Hemati zeinab. Mothers’ challenges after infants’ discharge from neonatal intensive care unit: A qualitative study. Iran J Neonatol IJN. 2017;(Online First). doi:10.22038/ijn.2017.15546.1174

8.	Jiang X, Jiang H, Shan SS, Huang R. Breastfeeding experience of postnatal mothers separated from preterm infants after discharge: a phenomenology qualitative approach. BMC Pregnancy Childbirth. 2024;24(1):28. doi:10.1186/s12884-023-06230-z

9.	Jonsdottir RB, Flacking R, Jonsdottir H. Breastfeeding initiation, duration, and experiences of mothers of late preterm twins: a mixed-methods study. Int Breastfeed J. 2022;17(1):68. doi:10.1186/s13006-022-00507-3

10.	Kair LR, Flaherman VJ, Newby KA, Colaizy TT. The experience of breastfeeding the late preterm infant: a qualitative study. Breastfeed Med Off J Acad Breastfeed Med. 2015;10(2):102–6. doi:10.1089/bfm.2014.0121

11.	Kennedy TS, Oakland MJ, Brotherson MJ. Making feeding decisions for preterm low birth weight infants: a family systems approach. Top Clin Nutr. 2000;15(2):38–84. doi:10.1097/00008486-200015020-00009

12.	Palmer L, Ericson J. A qualitative study on the breastfeeding experience of mothers of preterm infants in the first 12 months after birth. Int Breastfeed J. 2019;14(Journal Article):35. doi:10.1186/s13006-019-0229-6

13.	Parker MG, Hwang SS, Forbes ES, Colvin BN, Brown KR, Colson ER. Use of the Theory of Planned Behavior Framework to Understand Breastfeeding Decision-Making Among Mothers of Preterm Infants. Breastfeed Med Off J Acad Breastfeed Med. 2020;15(10):608–15. doi:10.1089/bfm.2020.0127

14.	Premji SS, Currie G, Reilly S, Dosani A, Oliver LM, Lodha AK, et al. A qualitative study: Mothers of late preterm infants relate their experiences of community-based care. PLoS ONE. 2017;12(3):1–13. doi:10.1371/journal.pone.0174419

15.	Reyna BA, Pickler RH, Thompson A. A descriptive study of mothers’ experiences feeding their preterm infants after discharge. Adv Neonatal Care Off J Natl Assoc Neonatal Nurses. 2006;6(6):333–40. doi:10.1016/j.adnc.2006.08.007 PubMed PMID: 17208164; PubMed Central PMCID: PMC3646381.

16.	Valizadeh S, Penjvini S. Mothers’ Experiences of Breastfeeding Premature Infants after Discharge from NICU; A Qualitative Study. Indian J Appl Res. 2011;4(1):374–8. doi:10.15373/2249555X/JAN2014/111

17.	Wheeler BJ. Human-milk feeding after NICU discharge. Neonatal Netw. 2009 Nov;28(6):381–9. doi:10.1891/0730-0832.28.6.381

18.	Niela-Vilen H, Axelin A, Melender HL, Salantera S. Aiming to be a breastfeeding mother in a neonatal intensive care unit and at home: a thematic analysis of peer-support group discussion in social media. Matern Child Nutr. 2015;11(4):712–26. doi:10.1111/mcn.12108

19.	Brown LF, Griffin J, Reyna B, Lewis M. The development of a mother’s internal working model of feeding. J Spec Pediatr Nurs JSPN. 2013;18(1):54–64. doi:10.1111/jspn.12011

20.	Niela-Vilen H, Axelin A, Salantera S, Melender HL. A Typology of Breastfeeding Mothers of Preterm Infants: A Qualitative Analysis. Adv Neonatal Care. 2019;19(1):42–50. doi:10.1097/ANC.0000000000000542

21.	Marigheti Brassarola HG, Penariol Natarelli TR, Monti Fonseca LM. Use of the WhatsApp® group in the post-discharge follow-up of the premature infant: implications for nursing care. Anna Nery Sch J Nurs Esc Anna Nery Rev Enferm. 2003;27(Journal Article):1–9. doi:10.1590/2177-9465-EAN-2022-0205en

22.	Callen J, Pinelli J, Atkinson S, Saigal S. Qualitative analysis of barriers to breastfeeding in very-low-birthweight infants in the hospital and postdischarge. Adv Neonatal Care Elsevier Sci. 2005;5(2):93–103. doi:10.1016/j.adnc.2004.12.005
